# Two Novel Semisynthetic Lipoglycopeptides Active against *Staphylococcus aureus* Biofilms and Cells in Late Stationary Growth Phase

**DOI:** 10.3390/ph14111182

**Published:** 2021-11-19

**Authors:** Vladimir Vimberg, Leona Zieglerova, Aninda Mazumdar, Zsolt Szűcs, Aniko Borbás, Pál Herczegh, Gabriela Balikova Novotna

**Affiliations:** 1 Institute of Microbiology, Czech Academy of Sciences, Průmyslova 595, 25250 Vestec, Czech Republic; zieglerova.leona@seznam.cz (L.Z.); anindamazumdar@gmail.com (A.M.); gnovotna@biomed.cas.cz (G.B.N.); 2 Department of Pharmaceutical Chemistry, University of Debrecen, Egyetem Tér 1, H-4032 Debrecen, Hungary; szucs.zsolt@pharm.unideb.hu (Z.S.); borbas.aniko@pharm.unideb.hu (A.B.); herczeghp@gmail.com (P.H.)

**Keywords:** antibiotic resistance, glycopeptide antibiotics, *Staphylococcus aureus*, teicoplanin pseudoaglycon

## Abstract

The increase in antibiotic resistance among Gram-positive bacteria underscores the urgent need to develop new antibiotics. New antibiotics should target actively growing susceptible bacteria that are resistant to clinically accepted antibiotics including bacteria that are not growing or are protected in a biofilm environment. In this paper, we compare the in vitro activities of two new semisynthetic glycopeptide antibiotics, MA79 and ERJ390, with two clinically used glycopeptide antibiotics—vancomycin and teicoplanin. The new antibiotics effectively killed not only exponentially growing cells of *Staphylococcus aureus*, but also cells in the stationary growth phase and biofilm.

## 1. Introduction

**Figure 1:**
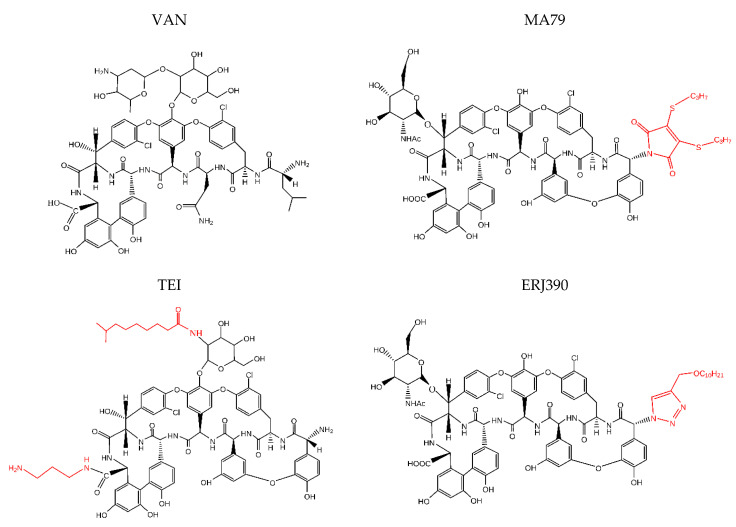
Chemical structures of the antibiotics: vancomycin (VAN), teicoplanin (TEI), MA79, and ERJ390. Lipophilic moieties are highlighted in red.

For many years, the glycopeptide antibiotics vancomycin (VAN) and teicoplanin (TEI) (Figure 1) were the only glycopeptide antibiotics used clinically to treat severe infections caused by methicillin-resistant *Staphylococcus aureus* (MRSA), currently recognized by the World Health Organization as one of the greatest threats to world health [1]. These antibiotics bind to the D-alanyl-D-alanine (D-Ala-D-Ala) terminus of the cell wall peptidoglycan precursor of the actively dividing bacterial cell. This interaction inhibits peptidoglycan polymerization, resulting in the disruption of cell wall synthesis. While vancomycin binds to the nascent peptidoglycan chain in a dimerized form, teicoplanin has been proposed to bind to the cell membrane lipid II substrate via its lipophilic acyl side chain, bringing the antibiotic in close proximity to the nascent peptidoglycan [2,3,4]. Vancomycin and teicoplanin are not active against non-dividing cells. Nonetheless, the transition of *S. aureus* from active growth to the non-dividing state induces the expression of a variety of virulence factors that facilitate bacterial invasion, local spread of infection [5], and biofilm formation; therefore, it is important to develop antibiotics that can act on non-dividing *S. aureus* cells.

Recently, we introduced a new group of glycopeptide derivatives in which the primary amino function of the teicoplanin pseudoaglycone was replaced [6,7,8]. Figure 1 shows maleimido teicoplanin pseudoaglycone with two propylthiol groups (MA79) and teicoplanin pseudoaglycone derivative with a lipophilic n-decyl chain (ERJ390), both of which exhibited in vitro activity against clinical isolates of *S. aureus*, coagulase-negative staphylococci (CoNS): *Staphylococcus epidermidis* and *Staphylococcus haemolyticus* (including biofilm-producing strains) [9].

The new glycopeptide antibiotics MA79 and ERJ390 were effective against vancomycin-intermediate *S. aureus* (VISA) and against CoNS resistant to teicoplanin. They also showed good efficacy against vanA- and vanB-type vancomycin-resistant enterococci (VRE). The vanA- and vanB-type resistance is mediated by the conversion of D-Ala-D-Ala-containing peptidoglycan precursors to D-alanine-D-lactate, which reduces the affinity of vancomycin and teicoplanin [4]. The preservation of MA79 and ERJ390 activity against VRE strains suggests that the binding of MA79 and ERJ390 to D-Ala-D-Ala is not the main antibiotic target [9]. The comparison of the potential of MA79 and ERJ390 to compete with fluorescently labeled vancomycin and teicoplanin bound to the cell wall of the *S. aureus* ATCC29213 strain confirmed that MA79 and ERJ390 are bound to the bacterial cell wall not only via D-Ala-D-Ala residues of the nascent peptidoglycan, but that the lipophilic substituents of MA79 and ERJ390 also enhanced the interaction of the antibiotics with the cell [9].

We hypothesized that the enhanced interaction of MA79 and ERJ390 with the bacterial cell wall could kill *S. aureus* cells even in the non-dividing state. Therefore, we compared the bacteriolytic properties of the antibiotics against *S. aureus* at different growth stages as well as in biofilm.

## 2. Results

### 2.1. MA79 and ERJ390 Are Bactericidal against Exponential and Stationary-Phase S. aureus

To investigate the bacteriolytic activity of the new lipoglycopeptide antibiotics MA79 and ERJ390, *S. aureus* strain 8325 was grown to the exponential or late stationary growth phase (see Figure S1) and then treated with either MA79, ERJ390, VAN, or TEI.

**Figure 2:**
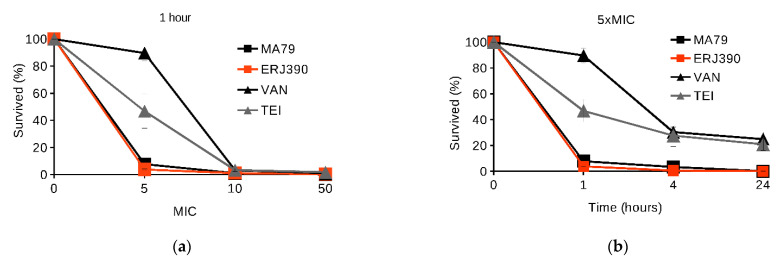
Killing of *S. aureus* 8325 cells in exponential phase by antibiotics vancomycin (VAN), teicoplanin (TEI), MA79, and ERJ390 at one hour after treatment of the bacteria with the antibiotics at various concentrations exceeding the MIC (**a**); or at time points after treatment of the bacteria with the antibiotics at a concentration exceeding the MIC 5-fold (**b**).

Firstly, we tested the ability of the antibiotics at concentrations 5, 10, and 50 times higher than the MIC (see Section 4) to kill *S. aureus* 8325 cells in one hour of incubation (see Figure 2a). MA79 and ERJ390 killed exponentially growing cells at concentrations that exceeded the MIC of the antibiotics by 5-fold (see Figure 2a). However, concentrations of VAN and TEI 10-fold higher than the corresponding MICs were required to kill exponentially growing cells in one hour (see Figure 2a).

Secondly, we tested the ability of the antibiotics at concentrations five times higher than the MICs to kill *S. aureus* 8325 cells after 1, 4, and 24 h of incubation with the antibiotics. MA79 and ERJ390 at concentrations five times higher than their MICs killed the cells in the exponential phase after one hour, while VAN and TEI could not completely sterilize the growth medium even after 24 h (see Figure 2b).

**Figure 3:**
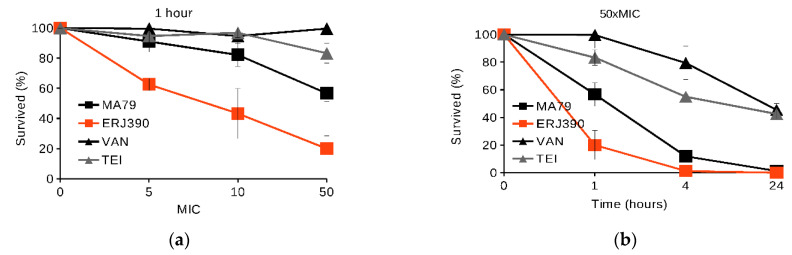
Killing of *S. aureus* 8325 cells in stationary phase by antibiotics vancomycin (VAN), teicoplanin (TEI), MA79, and ERJ390 at one hour after treatment of the bacteria with the antibiotics at various concentrations exceeding the MIC (**a**) and at time points after treatment of the bacteria with the antibiotics at a concentration exceeding the MIC 50-fold (**b**).

MA79 and ERJ390 were also more active than VAN and TEI in killing cells of *S. aureus* 8325 in the late stationary phase (see Figure 3). ERJ390 and MA79 killed approximately 80% and 40% of cells, respectively, within one hour at concentrations 50-fold higher than the MIC (see Figure 3a), whereas VAN and TEI were nearly inactive. After 24 h, both MA79 and ERJ390 at concentrations 50-fold higher than the MIC had completely sterilized the growth medium (see Figure 3b).

**Figure 4:**
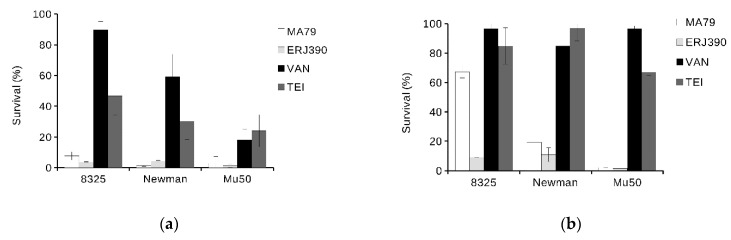
Killing of cells of different *S. aureus* strains during one hour of treatment by antibiotics vancomycin (VAN), teicoplanin (TEI), MA79, and ERJ390 pre-grown to exponential (**a**) or stationary (**b**) growth phases. The antibiotics were used at a concentration exceeding the MIC 5-fold and 50-fold against *S. aureus* cells in exponential or stationary growth phases, respectively.

Antibiotics MA79 and ERJ390 were also bactericidal against the exponential and stationary phase cells of vancomycin-susceptible *S. aureus* Newman and vancomycin-intermediate *S. aureus* Mu50 strains (see Figure 4). ERJ390 was the most active compound against all *S. aureus* strains tested.

### 2.2. MA79 and ERJ390 Do Not Induce S. aureus Biofilm Formation

**Figure 5:**
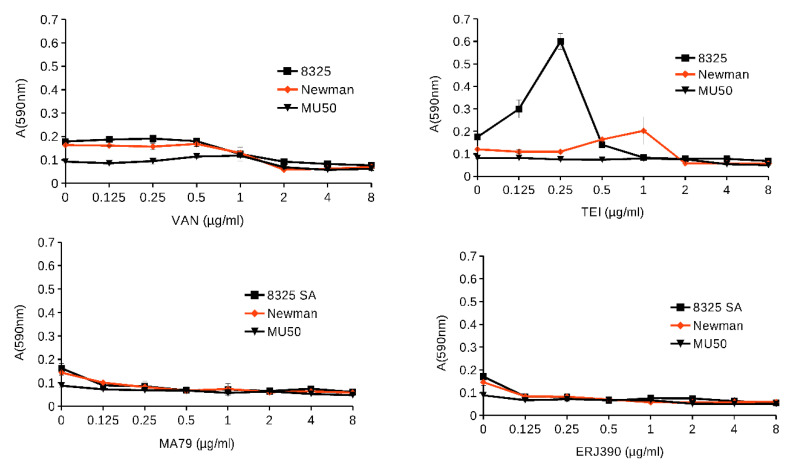
Biofilm formation of *S. aureus* in the presence of different concentrations of the VAN, TEI, MA79, and ERJ390.

Antibiotics at subinhibitory concentrations can induce biofilm formation in bacteria [10]. Therefore, the ability of MA79 and ERJ390 to induce biofilm formation in *S. aureus* strains 8325, Newman, and Mu50 was investigated. Under our experimental conditions, biofilm formation was not induced or abrogated at subinhibitory concentrations of VAN [11]. On the contrary, TEI strongly induced biofilm formation in 8325 and to a lesser extent in Newman (Figure 5). Mu50 did not form biofilm under our growth conditions. MA79 and ERJ390 inhibited biofilm formation at subinhibitory concentrations. Thus, MA79 and ERJ390 did not induce *S. aureus* biofilm formation but prevented it at subinhibitory concentrations.

### 2.3. ERJ390 Eradicates S. aureus Biofilm

**Figure 6:**
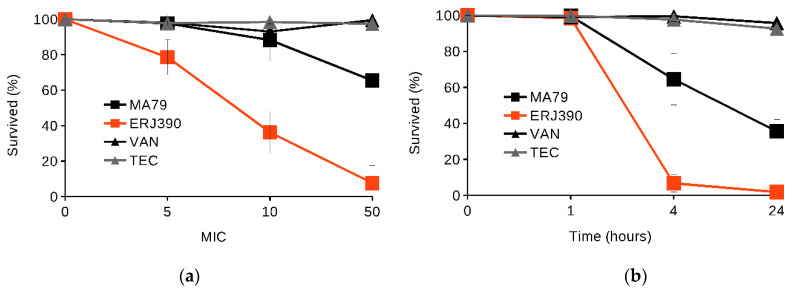
Killing of *S. aureus* cells in biofilm at different concentrations of antibiotics above MICs after four hours of incubation (**a**). Killing of *S. aureus* cells in biofilm at concentrations of the antibiotics 50 times higher than the MICs after different incubation times (**b**).

The treatment of *S. aureus* biofilms is challenging because even the most potent antibiotics have little effect on established biofilms [12,13,14]. In the present study, biofilms of *S. aureus* 8325 established overnight were treated with MA79, ERJ390, VAN, or TEI and cell viability was monitored by MTT staining (see Figure 6). MTT assay was employed for the quantification of biofilm biomass and metabolic activity [15]. MTT is a yellowish aqueous solution and yields a water-insoluble violet-blue formazan that is formed due to reduction by dehydrogenases and reducing agents present in metabolically active cells. Biofilm metabolic activity has been previously shown to be proportional to the production of formazan by measuring absorbance at A_450nm_ [15].

While VAN and TEI were unable to kill the bacteria in the biofilm, MA79 killed about 30% of the bacteria at a concentration 50 times higher than the MIC, and ERJ390 killed almost all the cells in the biofilm after four hours of incubation with the antibiotic (see Figure 6a). After extending the antibiotic treatment to 24 h, all bacterial cells in the biofilm were killed by ERJ390 and 60% of the cells were killed by MA79 (see Figure 6b). Therefore, this result shows the potential of the novel glycopeptide antibiotics to eradicate biofilms.

### 2.4. MA79 and ERJ390 Do Not Cause Resistance Selection

**Figure 7:**
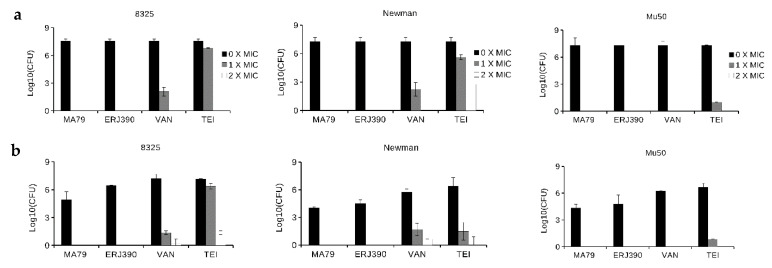
Selection of *S. aureus* 8325, Newman, and Mu50 resistant mutants, with (**a**) or without (**b**) prior incubation with subinhibitory concentration of antibiotics. The graphs show the number of colonies that appeared on the BHI agar plates with the respective antibiotic at 0-fold, 1-fold, or 2-fold MIC.

The development of resistance to commonly used antibiotics is the major drawback of antibiotic use [16]. We investigated the potential of MA79 and ERJ390 to select resistant mutants in *S. aureus*. No colonies appeared on plates containing MA79 or ERJ390, regardless of whether the bacteria were pre-incubated with sub-inhibitory concentrations of the respective antibiotics (see Figure 7a,b). No mutants with elevated MICs of MA79 or ERJ390 were selected even after 10 days of bacterial growth in the presence of subinhibitory concentrations of the antibiotics.

## 3. Discussion

In the last decade, the modification of the glycopeptide antibiotics vancomycin, teicoplanin, and ristocetin with different side chains, including hydrophobic and lipophilic moieties, have yielded new antibiotics that exhibit enhanced antibacterial activity and, in certain cases, high and broad-spectrum antiviral activity [17]. Three semi-synthetic lipoglycopeptide antibiotics, namely, telavancin, dalbavancin, and oritavancin, were approved for clinical use. Nevertheless, research has been conducted to further improve glycopeptide antibiotics [18]. Maleimido teicoplanin pseudoaglycone with two propylthiol groups, MA79, and teicoplanin pseudoaglycone derivative with a lipophilic n-decyl chain, ERJ390 (see Figure 1), have both been reported as effective antibiotics against various Gram-positive bacteria, including bacteria highly resistant to vancomycin and teicoplanin [9]. However, a more detailed review of the antimicrobial activity of these antibiotics was lacking.

Our present experimental data demonstrate the bactericidal activity of MA79 and especially ERJ390 against *S. aureus* cells in the exponential and late stationary phase. The semisynthetic lipoglycopeptide antibiotic oritavancin, recently approved for clinical use, kills nondividing *S. aureus* cells, whereas VAN and dalbavancin do not [19,20]. The bacteriolytic activity of oritavancin against *S. aureus* cells in the stationary growth phase was effective at a concentration that exceeded the MIC by more than 100–200-fold. This is a much higher concentration than in the case of MA79 and ERJ390. Thus, the bacteriolytic activity of MA79 and ERJ390 appears to be equal to or even higher than that demonstrated for oritavancin [19]. Moreover, ERJ390 is more efficient than MA79 (Figure 2 and Figure 3).

The killing of *S. aureus* cells in biofilm has been demonstrated for telavancin, oritavancin, and dalbavancin [12,21,22]. However, none of the antibiotics were able to completely kill the bacterial cells within the biofilms, even at concentrations exceeding 100 times the MIC. In comparison, ERJ390 was more efficient in killing bacterial cells within the biofilm and was able to kill all bacterial cells in the biofilm within 24 h at concentrations greater than 50 times the MIC (see Figure 6).

The observed activity of ERJ390 and MA79 against *S. aureus* cells in the stationary growth phase and in biofilms can be explained by the ability of the antibiotics to bind to the membrane. For oritavancin and telavancin, it has been demonstrated that their lipophilic side chains can anchor the antibiotics to bacterial membranes, leading to membrane depolarization and disruption, thus facilitating the bacteriolytic activity of glycopeptide antibiotics against non-dividing bacterial cells [17,23,24,25]. ERJ390 possessed a stronger killing activity than MA79. The interaction of ERJ390 with the membrane could be stronger than that of MA79, which was supported by the increased ability of ERJ390 to supplant fluorescently labeled teicoplanin from the cells [9]. This could explain the high killing efficiency of ERJ390.

The increased activity of ERJ390 may also be explained by the self-aggregation of the glycopeptide antibiotic [7]. It is known that self-aggregation enhances the interaction of oritavancin with the membrane and increases the bacteriolytic activity [26]. Self-aggregation of MA79 was not reported, which partly explains the lower activity of MA79 in killing non-dividing cells.

It is known that VAN, TEI, and dalbavancin can select *S. aureus* mutants with 16–128-fold increased resistance by multiple incubation steps with the antibiotics [27,28]. The resistance selection potential of MA79 and ERJ390 was not observed, suggesting that these antibiotics may be safe in terms of resistance development. The only reduction in susceptibility to MA79 and ERJ390 reported to date was observed in *S. aureus* expressing the membrane protein VanZ [29]. The expression of VanZ reduced the susceptibility of *S. aureus* to all lipoglycopeptide antibiotics. The acquisition and spread of VanZ genes in *S. aureus* may become a problem in the future. The encoding *vanZ* gene, as part of the *vanA* gene cluster, has occasionally been transferred to *S. aureus* by transposons from enterococci, resulting in highly vancomycin-resistant strains [30]. Although the frequency of such an event appears to be low due to the high fitness cost of *vanHAX*-mediated resistance in *S. aureus*, vancomycin-resistant staphylococcal strains may be precursors for the generation of *vanZ*-bearing mobile genetic elements that can interfere with the action of semisynthetic lipoglycopeptide antibiotics.

## 4. Materials and Methods

### 4.1. Strains

*S. aureus* strains 8325, Mu50, and Newman were kindly provided by Dr. Malcolm J Horsburgh. *S. aureus* strain 8325 [31] is considered as the reference genome in the NCBI database (MIC_VAN_ = 1 µg/mL; MIC_TEI_ = 2 µg/mL; MIC_MA79_ = 0.5 µg/mL; MIC_ERJ390_ = 0.25 µg/mL). Mu50 was the first MRSA strain with vancomycin resistance isolated in 1997 [32] (MIC_VAN_ = 4 µg/mL; MIC_TEI_ = 4 µg/mL; MIC_MA79_ = 0.5 µg/mL; MIC_ERJ390_ = 0.25 µg/mL). *S. aureus* strain Newman [33] (MIC_VAN_ = 0.5 µg/mL; MIC_TEI_ = 0.25 µg/mL; MIC_MA79_ = 0.5 µg/mL; MIC_ERJ390_ = 0.25 µg/mL) was isolated in 1952 from a human infection and displays robust virulence properties in animal models of disease and has already been extensively analyzed for its molecular traits of staphylococcal pathogenesis. The absence of drug resistance genes reflects the general antibiotic-susceptible phenotype of *S. aureus* Newman. *S. aureus* strains were pre-grown overnight on brain–heart infusion (BHI) agar plates at 37 °C prior to the experiments.

### 4.2. Antibiotics

Vancomycin (V2002) and teicoplanin (T0578) were bought from Sigma-Aldrich^®^ (St. Louis, MO, USA). MA79 and ERJ390 were synthesized as previously described [6,7]. For each experiment, new stock solutions of antibiotics were prepared.

**Figure 8:**
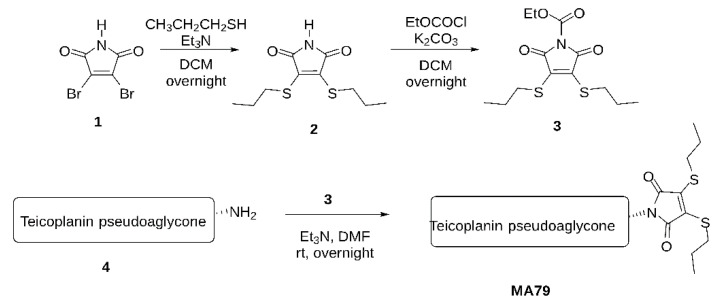
Schematic representation of MA79 synthesis.

The MA79 synthesis protocol is shown in Figure 8.

A. Maleimide bis-*n*-propylsulfide (2)

To a stirred solution of 2,3-dibromomaleimide (**1**) (510 mg, 2.0 mmol) in CH_2_Cl_2_ (30 mL), Et_3_N (4.0 mmol) and propyl mercaptane (380 μL, 4.2 mmol) were added under argon atmosphere and stirred for 3 h at room temperature. The reaction mixture was evaporated, and the crude product was purified by flash silica gel chromatography in *n*-hexane:ethyl acetate = 9:1 to give **2** (430 mg, 87%) as a yellow syrup. ^1^H NMR (400 MHz, CDCl_3_) δ 7.77 (1H, s, N*H*), 3.28–3.25 (4H, m, 2 × SC*H*_2_), 1.73–1.66 (4H, m, 2 × C*H*_2_), 1.06–1.02 (6H, m, 2 × C*H*_3_); ^13^C NMR (100 MHz, CDCl_3_) δ 166.3 (2C, 2 × C=O), 137.2 (2C, C=C), 33.6 (2C, 2 × *C*H_2_), 23.8 (2C, 2 × S*C*H_2_), 13.1 (2C, 2 × *C*H_3_); Analysis Calculated for C_10_H_15_NO_2_S_2_ C 48.95, H 6.16, N 5.71, O 13.04, S 26.14. Found: C 48.18, H 5.70, S 26.01.

B. *N*-Ethoxycarbonyl maleimide bis-*n*-propylsulfide (3)

To a stirred solution of maleimide bis-sulfide **2** (1.0 mmol) in dry acetone (20 mL), K_2_CO_3_ (1.2 mmol) and ethyl chloroformate (1.2 mmol) were added under argon atmosphere and stirred for 3 h at room temperature. The reaction mixture was diluted with CH_2_Cl_2_, filtered through a pad of Celite, and evaporated. The crude product was used for further steps without purification.

C. MA79

To a stirred solution of teicoplanin pseudoaglycone (140 mg, 0.1 mmol) in dry DMF (5 mL), *N*-ethoxycarbonyl maleimide bis-sulfides **3** (40 mg, 0.14 mmol) and Et_3_N (0.1 mmol) were added under argon atmosphere and stirred overnight at room temperature. The reaction mixture was evaporated, and the crude product was purified by silica gel chromatography in toluene:methanol = 9:1 to give **8d** (110 mg, 66%) as a yellow powder. MALDI-TOF MS: [M+Na]^+^ = 1651.02 m/z. Calculated for C_76_H_70_Cl_2_N_8_O_25_S_2_Na 1651.32 m/z.

**Figure 9:**
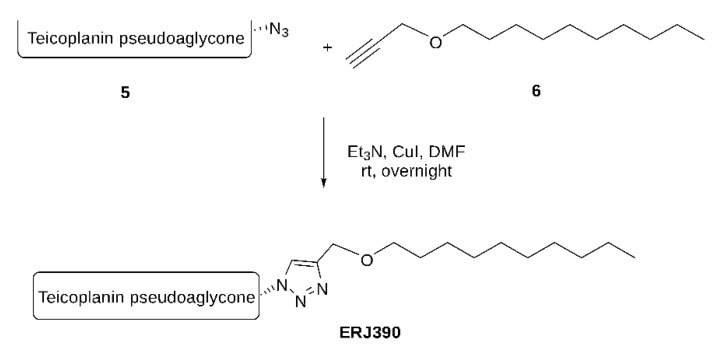
Schematic representation of the ERJ390 synthesis.

See Figure 9 for the ERJ390 synthesis protocol.

To a stirred solution of teicoplanin pseudoaglycone azide (140 mg, 0.1 mmol) in dry DMF (5 mL), *n*-decyl propargyl ether **6** (24 mg, 0.13 mmol), Et_3_N (1.0 equiv.), and Cu(I)I (20–30 mol%) were added under an argon atmosphere and stirred for overnight at room temperature. The reaction mixture was concentrated, and the crude product was purified by flash chromatography in toluene: MeOH = 1:1. (+1.0 *v*/*v*% acetic acid). Yield: 115 mg, 71%.

### 4.3. Susceptibility Testing

Minimum inhibitory concentrations (MICs) were determined by the microbroth dilution method in Mueller–Hinton (MH) medium according to the EUCAST guidelines (ISO 20776). Overnight cultures of *S. aureus* pre-grown on BHI agar at 37 °C were resuspended in 0.9% NaCl solution to McFarland = 0.5. Five microliters of the resuspended *S. aureus* cells were pipetted into each well of a 96-well plate (Thermo Scientific^™^ 130188 BioLite 96 Well Multidish, Waltham, MA, USA) containing MH medium with concentration of antibiotics. Ranges of antibiotic concentrations tested were 0.0156–1024 µg/mL. Absorbance was measured at A_600nm_, using a BioTek^®^ Synergy HT spectrophotometer after 24 h of bacteria incubation in MH medium in 96-well plates at 37 °C. Each MIC test was performed three times in triplicate. Bacterial growth was considered to be completely inhibited by the antibiotic if the absorbance value was lower than the highest absorbance value of the wells containing only growth medium.

### 4.4. S. aureus Killing in Exponential and Late Stationary Growth Phases by the Antibiotics

*S. aureus* 8325 was pre-grown in MH medium at 37 °C to A_600nm_ = 0.6 or grown for 18 h until stationary phase was reached (see Figure S1). Glycopeptide antibiotics were added to the pre-cultured bacteria at concentrations 5, 10, and 50 times higher than the respective MICs and incubated at 37 °C for 1 h. Incubated bacteria without antibiotic were used as negative controls. The number of colony-forming units (CFUs) in antibiotic-treated or untreated samples was determined by spreading 10 µL of cultures diluted 10^1^–10^8^-fold in 0.9% NaCl on brain–heart infusion (BHI) agar plates. The number of CFUs in the untreated sample was set as 100% of the surviving bacterial cells. Each experiment was performed in triplicate and repeated three times. The time course of killing *S. aureus* 8325 in the exponential or stationary growth phase was performed at concentrations 5 and 50 times higher than the MIC, respectively, and analyzed after 0, 1, 4, and 24 h of incubation at 37 °C. Killing of *S. aureus* 8325, Newman, and Mu50 by antibiotics was analyzed after 1 h of incubation of the bacteria at 5 and 50-fold higher MICs for bacteria in the exponential and stationary growth phases, respectively.

### 4.5. S. aureus Biofilm Formation in the Presence of Glycopeptide Antibiotics

Biofilms of *S. aureus* were established in 96-well plates after 24-h bacterial growth in tryptic soy broth (TSB) at 37 °C [34] in the presence of the antibiotics: MA79, ERJ390, VAN, and TEI at concentrations of 0, 0.125, 0.25, 0.5, 1, 2, 4, and 8 µg/mL. After 24 h of incubation, the plates were carefully rinsed with water 3 times to remove non-adherent cells. Cells remaining in the wells were stained with 50 µL of 1% crystal violet for 15 min, then carefully rinsed with water 5 times and air dried. Crystal violet was recovered from the stained cells by adding 100 µL of a 96% ethanol solution. The absorbance of the plate was then measured at A_590nm_ using a TECAN Infinite Pro spectrophotometer. Each experiment was performed three times in triplicate.

### 4.6. Bacteria Killing in S. aureus Biofilm

The biofilm of *S. aureus* 8325 was established in 96-well plates after 24 h of bacterial growth in tryptic soy broth medium (TSB) at 37 °C (TSB) [34]. The biofilms were washed 3 times with 0.9% NaCl. Then, 100 µL of fresh TSB medium without or with antibiotics at concentrations 5, 10, and 50 times higher than the MICs was added to the biofilms. After incubating the biofilm with the antibiotics at 37 °C for 4 h, the biofilms were washed three times with 0.9% NaCl and then stained with 0.2% MTT for 1 h at 37 °C. The MTT assay was used to quantify biofilm biomass and metabolic activity [15]. The absorbance was measured at A_450nm_ using TECAN Infinite^®^ Pro spectrophotometer. Absorbance values of biofilms not treated with antibiotics were set as 100% of surviving cells. Each experiment was performed three times with four replicates each time. Killing of bacteria in the biofilm occurred at 1, 4, and 24 h when antibiotic concentrations were 50-fold higher than MICs.

### 4.7. Selection of Resistance

*S. aureus* strains 8325, Newman, and Mu50 were grown at 37 °C in 2 mL of MH medium to A_600nm_ = 0.6 in the absence or presence of half of MIC of MA79, ERJ390, VAN, or TEC. A total of 100 µL of pre-grown cells was plated on BHI agar plates with antibiotics at concentration 0, 1, or 2 times exceeding MICs. Colonies were counted after 24 h of incubation at 37 °C. The logarithmic values of numbers of appeared colonies (CFU) were compared. The experiment was repeated three times. Since we have not selected MA79- and ERJ390-resistant mutants by this approach, we increased the time of *S. aureus* incubation with half MIC of MA79 and ERJ390 for 10 days, diluting bacteria 100 times into fresh BHI medium with half MIC of the antibiotics every 12 h. MICs of the antibiotics against colonies that appeared on the plates in the presence of the highest antibiotic concentration were determined (see Table S1).

## 5. Conclusions

MA79 and ERJ390 are novel semisynthetic glycopeptides with improved activity against Gram-positive bacteria compared to VAN and TEC. They can overcome multiple glycopeptide resistance mechanisms and are significantly less susceptible to the development of resistance. The antibacterial activity of MA79 and ERJ390 is not solely dependent on interaction with D-Ala-D-Ala and could involve binding to the bacterial cell membrane. The mechanism of binding of the new glycopeptides to the cell membrane might be different from that of teicoplanin and dalbavancin. Further studies are required to discover the exact mechanisms of action of MA79 and ERJ390.

## Data Availability

Data is contained within the article and supplementary material.

## Supplementary Materials

The following are available online at https://www.mdpi.com/article/10.3390/ph14111182/s1, Figure S1: Growth curves of *Staphylococcus aureus* strains used in the study, Table S1: MICs of the antibiotics against wild-type (WT) *Staphylococcus aureus* strains and mutants selected in resistance selection experiments.
