# Supplementary figures and images for: Two Novel Semisynthetic Lipoglycopeptides Active against Staphylococcus aureus Biofilms and Cells in Late Stationary Growth Phase
Source: Pharmaceuticals (Basel). 2021 Nov 19;14(11):1182. doi: 10.3390/ph14111182 (PMC8619453; doi:10.3390/ph14111182)

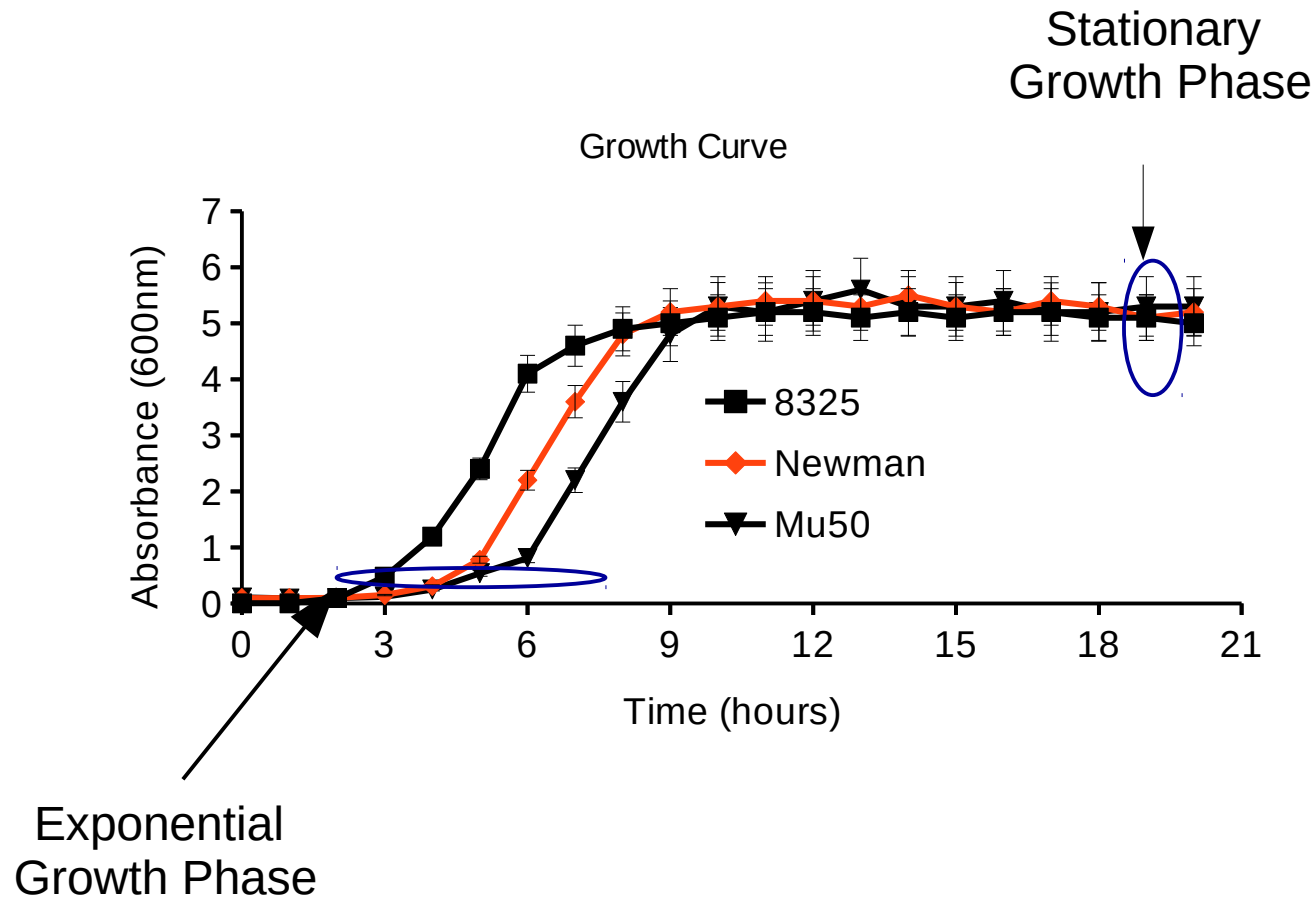

Supplement: Supplementary file 1 [file pharmaceuticals-14-01182-s001.zip › Figure_S1.pdf]
